# Therapeutic targets by traditional Chinese medicine for ischemia-reperfusion injury induced apoptosis on cardiovascular and cerebrovascular diseases

**DOI:** 10.3389/fphar.2022.934256

**Published:** 2022-08-19

**Authors:** Xiuli Cheng, Jin Hu, Xiaofeng Liu, Jonnea Japhet Tibenda, Xiaobo Wang, Qipeng Zhao

**Affiliations:** ^1^ Department of Pharmacy, People’s Hospital of Ningxia Hui Autonomous Region, Yinchuan, China; ^2^ Department of Preparation Center, General Hospital of Ningxia Medical University, Yinchuan, China; ^3^ School of Pharmacy, Ningxia Medical University, Yinchuan, China; ^4^ Research Institute of Integrated TCM and Western Medicine, Chengdu University of Traditional Chinese Medicine, Chengdu, China; ^5^ Key Laboratory of Ningxia Ethnomedicine Modernization, Ministry of Education (Ningxia Medical University), Yinchuan, China

**Keywords:** traditional Chinese medicine, cerebral ischemia, myocardial ischemia, apoptosis, molecular mechanisms

## Abstract

Traditional Chinese medicine (TCM) has a significant role in treating and preventing human diseases. Ischemic heart and cerebrovascular injuries are two types of diseases with different clinical manifestations with high prevalence and incidence. In recent years, it has been reported that many TCM has beneficial effects on ischemic diseases through the inhibition of apoptosis, which is the key target to treat myocardial and cerebral ischemia. This review provides a comprehensive summary of the mechanisms of various TCMs in treating ischemic cardiovascular and cerebrovascular diseases through anti-apoptotic targets and pathways. However, clinical investigations into elucidating the pharmacodynamic ingredients of TCM are still lacking, which should be further demystified in the future. Overall, the inhibition of apoptosis by TCM may be an effective strategy for treating ischemic cardio-cerebrovascular diseases.

## Graphical Abstract

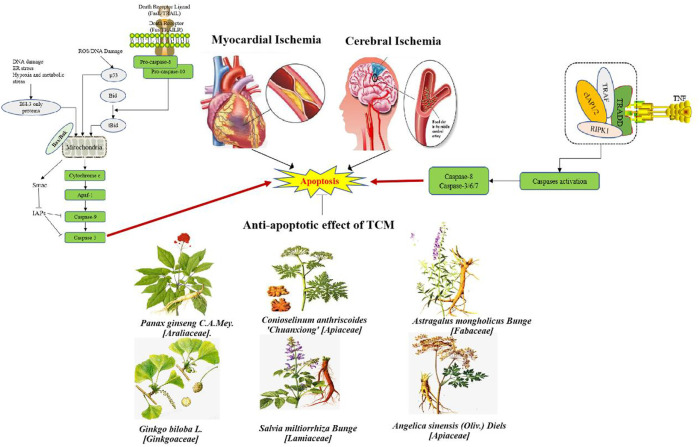

## 1 Introduction

Ischemic heart and cerebrovascular diseases have different clinical manifestations caused by insufficient local blood supply induced by vascular stenosis, atherosclerosis, or infarction (Battaglini et al., 2020). Modern medicine considers the heart and the brain closely related to regulating their corresponding functions through nerve reflexes and humoral coordination (Chen et al., 2017). Traditional Chinese medicine (TCM) argues that the pathogenesis of these two diseases is mainly caused by blood stasis and 

obstruction of blood flow (Yu et al., 2016). In other words, this is consistent with the holistic view and syndrome differentiation and treatment advocated by TCM (Luo et al., 2019). Herein, we choose cerebral ischemia and myocardial ischemia as the objects to explore the molecular mechanism of active ingredients identified from herbal medicines in treating these two diseases. Apoptosis is a naturally occurring homeostatic process to orchestrate cell death (Argüelles et al., 2019). An array of evidence has shown that ischemic myocardial, and brain injury is accompanied by extensive apoptosis, evidenced by cell blebbing and shrinkage, nuclear fragmentation, condensation, fragmentation of chromatin, and the formation of small vesicles (Gong et al., 2017; Dong et al., 2019; Xu et al., 2019). Hence, inhibiting excessive apoptosis is an effective strategy for treating these two diseases under ischemia.

**FIGURE 1:**
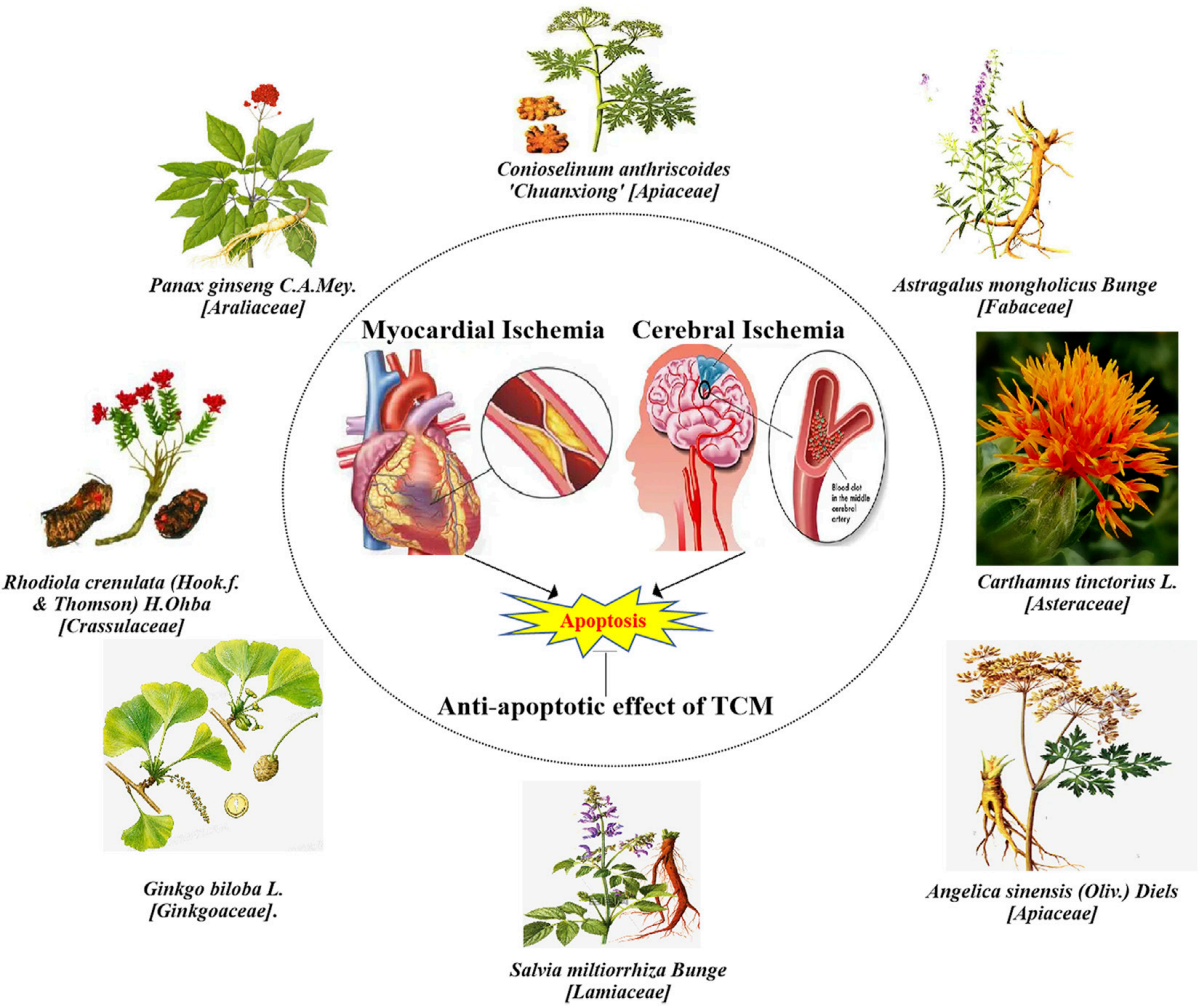
The representative Chinese herbs in treating myocardial ischemia and cerebral ischemia. Most herbs promote blood circulation and remove blood stasis, such as *Panax ginseng C.A.Mey.* [*Araliaceae*], *Astragalus mongholicus Bunge [Fabaceae], Conioselinum anthriscoides [Apiaceae], Rhodiola crenulata (Hook.f. & Thomson) H. Ohba* [*Crassulaceae*], *Carthamus tinctorius L.* [*Asteraceae*], *Ginkgo biloba L.* [*Ginkgoaceae*], *Angelica sinensis* (*Oliv.*) *Diels* [*Apiaceae*] and *Salvia miltiorrhiza Bunge* [*Lamiaceae*] can treat ischemic cardio-cerebrovascular injury and various complications through anti-apoptosis.

The therapeutic effect of TCM has cast a new light on cardiovascular and cerebrovascular diseases by inhibiting apoptosis, characterized by multiple components and targets (Wang et al., 2020a; Wang et al., 2021; Xie et al., 2021). However, anti-apoptosis’s molecular mechanism of active ingredients has not been fully reported. Therefore, this review aims to provide an individual research basis for treating different diseases by summarizing and analyzing the apoptosis-relevant molecular mechanism of TCM in treating cerebral and myocardial ischemia. The ischemic cardio-cerebrovascular protective effect of some TCM was shown in Figure 1.

## 2 Traditional Chinese medicine on myocardial and cerebral ischemia by inhibiting apoptosis

**FIGURE 2:**
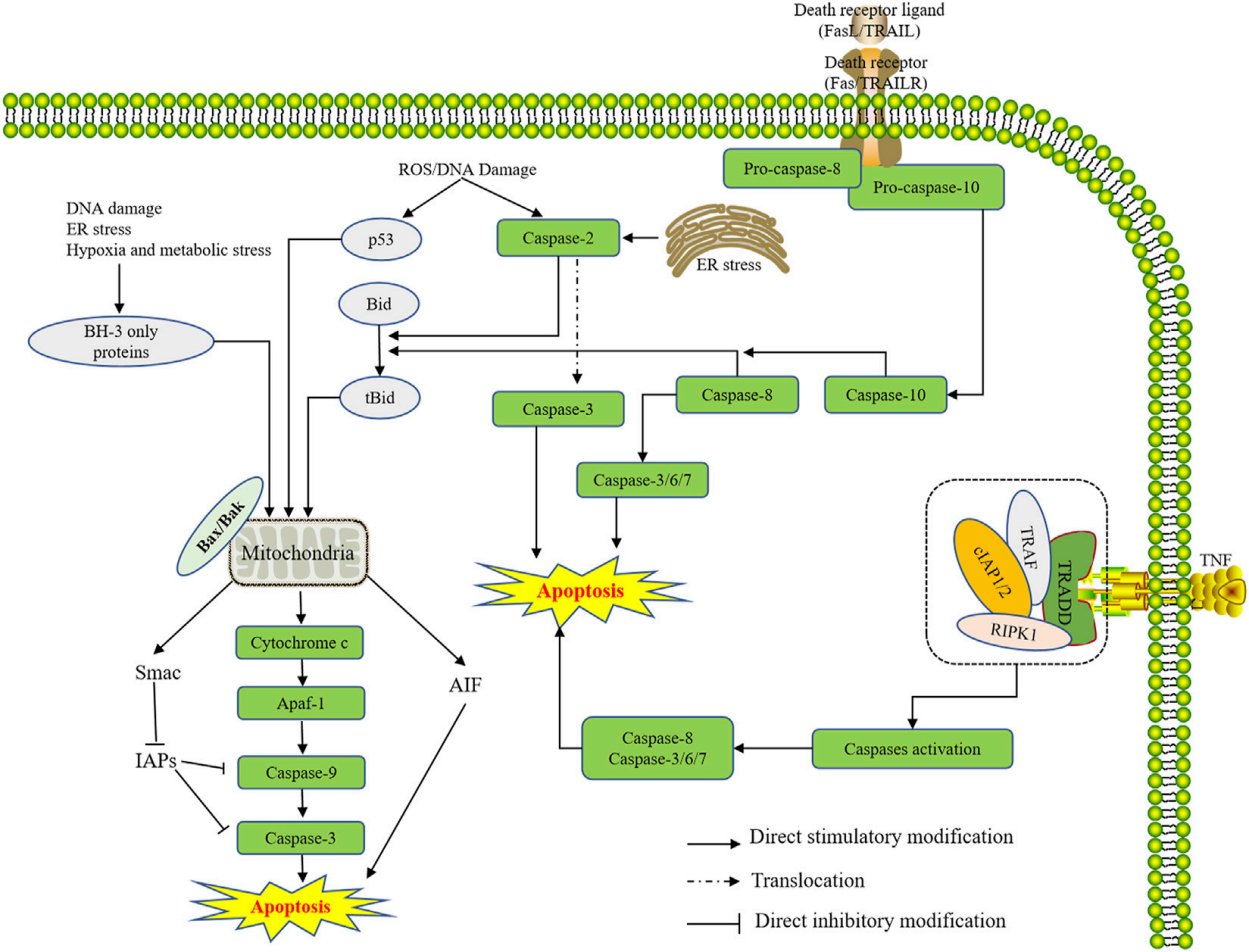
Endogenous and exogenous apoptotic events are involved in ischemic cardio-brain injuries. Major apoptosis pathways include mitochondria-dependent and death receptor-mediated. These apoptosis pathways together interacted mutually when ischemic diseases occurred. For the former, hypoxia-induced ROS surge and DNA damage directly activated caspase-2 and downstream Bid-activated mitochondrial apoptosis. In parallel, the activated p53 also stimulated the mitochondrial apoptotic process. Meanwhile, DNA damage, ER stress and hypoxia metabolic stress can evoke the mitochondrial apoptosis pathway by activating BH-3 only protein. As for the specific process of mitochondrial apoptosis, Bax and Bak dimers induced by hypoxia formed the channels of cyt-c and AIF in and out on the mitochondrial membrane, which further infuriated the overexpression of Apaf-1, caspase-9 and the apoptosis executor caspase-3 to seduce apoptosis. On the contrary, the increased Smac could negatively inhibit IAPs to further subdue mitochondrial apoptosis. For the latter, pro-caspase-8 and pro-caspase-10 could be activated by the combination of death receptors (FasL/TRAIL) and death ligands (Fas/TRAILR). Together with ER stress and caspase-2, caspase-3/6/7 was thus triggered to result in apoptosis. On the other hand, the formation of TRADD, TRAF, CIAP1/2 and RIPK1 complexes activated by TNF can also lead to apoptosis by irritating caspase-3/6/7/8.

Lately, studies have illustrated that apoptosis played an indispensable role in the pathophysiologic changes induced by ischemia (Jin et al., 2020). During prolonged ischemia, the decrease of intracellular pH and ATP caused by anaerobic metabolism leads to lactate accumulation. Despite the O_2_ level recovers after reperfusion, a surge generation of reactive oxygen species (ROS) occurs with neutrophil infiltration in ischemic tissues, which could activate a variety of molecular signaling pathways and ultimately lead to cell death by releasing proapoptotic proteins such as Cyt-c (Yadav et al., 2020). Mitochondrial permeability transition pore (MPTP) opening is another key point in cardiac I/R injury on account of its increased sensitivity to Ca^2+^ in the rat hypoxia/reoxygenation (H/R) myocardial model (Zhao et al., 2020). It could increase the expression of apoptosis-associated proteins (Cyt-c and Apaf 1), and activate caspase-3 and caspase-9, thereby leading to cardiomyocyte apoptosis (Yuan et al., 2019; Li et al., 2020a). TNF-α exerts its activity in cardiovascular pathophysiology through binding to its receptors, TNFR1 and TNFR2. While TNFR -associated factor 2 (TRAF2) plays a protective role in cardiac ischemia, its expression in the heart is induced along with pressure overload and could alleviate myocardial infarction (Li et al., 2019). The reported study explains that neuronal apoptosis may depend on the Fas/FasL pathway. Particularly, Fas/FasL is also involved in the oxygen-glucose deprivation/reperfusion model-induced Golgi fragmentation and apoptosis (Zhang et al., 2016). During myocardial ischemia-reperfusion injury (MIRI), the concentration of intracellular Ca^2+^ continues to increase, which leads to myocardial cell apoptosis and ultimately causes myocardial damage (Hu et al., 2020). Increasing evidence shows that ER stress contributes to I/R-induced damage (Lin, 2015). Furthermore, previous studies have shown that the hippocampal neurons’ apoptosis and apoptosis-related proteins such as CHOP, caspase-12, and GRP78 increased after I/R injury in rats (Sanderson et al., 2015). Studies mentioned above indicate that different apoptotic pathways played a crucial role in cerebral and myocardial ischemia. The research acknowledged that the mitochondrial apoptosis pathway plays a significant role in ischemic disease. The death receptor apoptosis pathway and endoplasmic reticulum apoptosis pathway are also important. These apoptosis pathways interacted mutually when ischemic disease occurred, ultimately leading to the cascade reaction of apoptosis, thereby aggravating the clinical symptoms of cardiovascular and cerebrovascular diseases. More details referring to these pathways are shown in Figure 2.

### 2.1 Treatment of traditional Chinese medicine on myocardial ischemia

#### 2.1.1 Chinese herbal extracts and drug pairs

The extracts of *Salvia miltiorrhiza Bunge* [Lamiaceae] (SM) and *Dalbergia odorifera T.C.Chen* [Fabaceae] (DO) have long been used in the treatment of myocardial ischemia and other cardiovascular diseases. SM and DO (SM-DO) volatile oil could increase the Bcl-2/Bax ratio in the chronic myocardial ischemia model (Lin et al., 2018). The combination of SM and *Carthamus tinctorius L. [Asteraceae]* (CT) could promote blood circulation and remove blood stasis. It can significantly inhibit the high expression of Bax and NF-κB p65 protein and induce the low expression of Bcl-2 protein in acute myocardial ischemia rats (Wang et al., 2019). *Aquilaria malaccensis Lam. [Thymelaeaceae]* (AM) alcohol extract exhibited protective effects on MI. The mechanism was mainly associated with the upregulation of Nrf2-ARE and the downregulation of Bcl-2 (Wang et al., 2020b). Furthermore, Zang et al. (2021) discovered that volatile oil of the *Acorus calamus L. [Acoraceae]* (AC) could inhibit apoptosis caused by myocardial infarction via down-regulating COX-2 and up-regulating PPAR protein. They activated vascular endothelial growth factor (VEGF) and cAMP signal pathways.

#### 2.1.2 Chinese medicinal formulae and injection

Xuan Bi Tong Yu Fang (XBTYF) is generally used to treat angina pectoris and coronary heart disease by decreasing the expression of caspase 3, Notch1, Bax, caspase 9, Dll4, and Cyt-c, however increasing the expression of VEGF and Bcl2 (Li et al., 2020b). Huoxin Pill (HXP) could improve cardiac function in MI mice by suppressing cardiac apoptosis and fibrosis partially through the suppression of the p53/Bax/Bcl-2 and TGF-β1/Smad2/3 pathways (Shen et al., 2020). Tongmai Yangxin pill (TMYX) is often used to treat coronary heart disease, irregular heartbeat and chest pain. The potential mechanism may be related to enhancing the activity of eNOS and the level of eNOS Ser (1177) phosphorylation in NR myocardium, increasing the Bcl-2 expression, while decreasing the caspase-3 and Bax expression, thus inhibiting cardiomyocyte apoptosis (Chen et al., 2020a). Gualou Xiebai Pinellia (GLXP) decoction has long been used to treat cardiovascular disease, which could protect the heart against I/R-induced myocardial blood flow decrease, myocardial infarction and apoptosis (Yan et al., 2018). Guizhi Glycyrrhiza decoction (GGD) is known for treating MIRI, arrhythmia, and other cardiovascular diseases using enhancing Bcl-2, PPAR-α, and PPAR-γ while reducing Bax, caspase-3 and caspase-9, hence inhibiting myocardial apoptosis. Furthermore, GGD can protect the myocardium from I/R injury by inhibiting the TLR4/NF-κB signal pathway, reducing the inflammatory response and cardiomyocyte apoptosis (Gao et al., 2019). Dried ginger-aconite decoction (DGAD) serves a therapeutic role in anti-MIRI via activating the PI3K/Akt/GSK-3β signal pathway to attenuate mitochondrial hypoxia injury and cardiomyocyte apoptosis (Xie et al., 2021). Qili Qiangxin capsule (QQX) has been verified to improve the mitochondrial-dependent apoptosis of cardiomyocytes induced by oxidative stress through the PI3K/AKT/GSK3β signal pathway (Zhao et al., 2019). The therapeutic effect of Qishen granule (QSG) on myocardial ischemia was to inhibit cardiomyocyte apoptosis by increasing the levels of Bcl-2 and caspase-3/12 and decreasing the expression of Bax and cleaved caspase-3/12 (Zhang et al., 2020a). Huang et al. previously determined that SM and ligustrazine injection (SLI) could relieve I/R injury in cardiomyocytes and inhibit apoptosis through activating the Akt-eNOS signaling pathway and down-regulating the expression of proapoptotic factors (Huang et al., 2016). Danhong injection (DHI) could mitigate inflammation associated with MI through inhibiting NF-κB nuclear translocation and regulating miRNAs, thus improving cardiac function in myocardial infarction rats (Chen et al., 2019a). Shengmai injection (SMI) could increase cell viability, prevent cell apoptosis, and increase PI3K and p-Akt expression in H9c2 cardiomyocytes injured by doxorubicin (Li et al., 2020c). Furthermore, it has been reported that the SMI-driven reduction in apoptosis was related to the change in the Bcl-2/Bax ratio (Liu et al., 2018a). Some studies indicated that Shuxuening injection (SXNI) inhibited Bax/Bcl-2 and blocked caspase-3 activation expression from CIRI-induced hippocampal neuronal apoptosis (Li et al., 2021). The beneficial effect of Hongjingtian injection (HJTI) on myocardial I/R is to improve mitochondrial function, regulate autophagy, and inhibit apoptosis through the AMPK/mTOR pathway. It can significantly reduce the cleaved caspase-3 and increase the Bcl-2/Bax ratio (Zhao et al., 2021).

### 2.2 Active ingredients of traditional Chinese medicine

Tanshinone IIA is a fat-soluble diterpene extracted from SM and has the effect of reducing myocardial ischemic injury. It can increase the viability and inhibit the apoptosis of injured cardiomyocytes by enhancing the expression of Bcl-2 and Bak, whereas lowering the expression of Cyt-c, caspase-3 and Apaf-1 in myocardial tissue. In addition, it can regulate mitochondrial function through PI3K/Akt/Bad pathway and reduce cardiomyocyte apoptosis in obese rats combined with cyclosporine A (Wu et al., 2019; Tai et al., 2021). Salvianolic acid A (Sal-A) and salvianolic acid B (Sal-B) have predominant pharmacological activities as the active components of SM, Sal-A treatment could decrease tunnel-positive cells and pro-apoptotic Bax after MI. Further study indicated that it could promote thioredoxin and inhibit the activation of c-jun N-terminal kinase (JNK) from attenuating inflammation and apoptosis after MI (Zhou et al., 2020). Sal-B could relieve MIRI, ameliorate cardiac function, and reduce myocardial infarct size by means of activating PI3K/Akt expression and inhibiting HMGB1 expression (Liu et al., 2020). As major ingredients of *Panax ginseng C.A.Mey. [Araliaceae]*, ginsenoside Rg1 (G-Rg1) and ginsenoside-Rb3 (G-Rb3) have benefited the heart condition. Li et al. (2018b) depicted the protective potential of G-Rg1 against MIRI with the mechanism of suppressing myocardial apoptosis and regulating energy metabolism. G-Rb3 preconditioning inhibited the accumulation of intracellular ROS and partially saved hypoxia/reoxygenation caused by oxidative stress and apoptosis in cardiomyocytes (Chen et al., 2019b; Sun et al., 2019). 6-gingerol could remarkably inhibit cardiomyocyte apoptosis and caspase-3 activation induced by I/R. It can also increase the expression of PI3K, p-Akt and Akt in myocardial tissue (Lv et al., 2018). It has been confirmed that Salidroside could reduce myocardial inflammation and apoptosis and promote angiogenesis through up-regulating the expression of Akt, Bcl-2, VEGF, and eNOS whereas down-regulating the expression of IL-1β, TNF-α, TGF-β1 and Bax (Chen et al., 2019c). Salidroside pretreatment can also attenuate apoptosis in the I/R injury model induced by ER stress by decreasing cleaved caspase-12 and Bax and the activity of caspase-3 while increasing the expression of Bcl-2 (Sun et al., 2018). Paeoniflorin has been verified that it could significantly increase the Bcl-2 protein levels and decrease the p-jnk, caspase3, Bax-p-erk and p-p38 protein induced by MIRI (Wu et al., 2020). Total paeony glycoside substantially increased cell viability time- and dose-dependent. In addition, Total paeony glycoside may reduce the apoptosis rate and oxidative stress of H9C2 cells by inhibiting the PI3K/Akt signal pathway (Shen et al., 2018). Paeonol could up-regulate the Bcl-2 expression and down-regulate the caspase-8, caspase-9, caspase-3 and PARP expression in the MIRI model (Tsai et al., 2021). Ginkgolide B has a protective effect on apoptosis caused by myocardial infarction and ischemia. The mechanism concentrates on inhibiting of Bax and cleaved caspase-3 and the enhancement of Bcl-2 (Ren et al., 2020). The molecular mechanisms of Chinese herbal prescriptions and monomer components in the treatment of myocardial ischemia by inhibiting apoptosis are shown in Table 1.

**TABLE 1:** The involved mechanisms of representative TCM prescriptions in the treatment of myocardial ischemia.

Categories	TCM	Composition	Mechanisms	References
Drug pairs	SM-DO	*Salvia miltiorrhiza Bunge [Lamiaceae]* and *the volatile oil of Dalbergia odorifera T.C.Chen [Fabaceae]*	Increasing expression of Bcl-2/Bax, Akt and GSK-3β	Lin et al. (2018)
	SM-CT	*Salvia miltiorrhiza Bunge [Lamiaceae]* and *Carthamus tinctorius L. [Asteraceae]*	Increasing expression of NF-κB p65 and Bcl-2/Bax	Wang et al. (2019)
Extract	AM	*Aquilaria malaccensis Lam. [Thymelaeaceae] alcohol extract*	Upregulating Nrf2-ARE; suppressing Bcl-2 pathway	Wang et al. (2020f)
	AC	*The volatile oil of Acorus calamus L. [Acoraceae]*	Downregulating the COX-2 protein; upregulating the PPAR-α protein	Zang et al. (2021)
Formulas	XBTYF	*Corydalis yanhusuo [Papaveraceae], Panax notoginseng (Burkill) [Araliaceae], Conioselinum anthriscoides [Apiaceae], Thespesia populnea [Malvaceae], Panax ginseng [Araliaceae]* and *Cinnamomum camphora [Lauraceae]*	Reducing the expression of Notch1, Dll4, Bax, caspase-3, caspase-9 and Cyt-c; increasing the expression of VEGF-A and Bcl-2	Li et al. (2020d)
	HXP	*Ganoderma lucidum, artificial Musk, Bear Bile, Carthamus tinctorius L. [Asteraceae], Bezoar, Hordeum vulgare L. [Poaceae], Panax ginseng. [Araliaceae], Borassus flabellifer L. [Arecaceae], Aconitum lethale Griff. [Ranunculaceae]* and *Cinnamomum camphora [Lauraceae]*	Decreasing the p53 and Bax/Bcl-2 protein expression	Shen et al. (2020)
	TMYX	*Rehmannia glutinosa [Orobanchaceae], Spatholobus suberectus [Fabaceae], Ophiopogon japonicus [Asparagaceae], Glycyrrhiza uralensis [Fabaceae], Reynoutria multiflora [Polygonaceae], donkey-hide glue, Schisandra chinensis [Schisandraceae], Codonopsis pilosula [Campanulaceae], Chinemys reevesii, Ziziphus jujuba [Rhamnaceae]* and *Neolitsea cassia. [Lauraceae]*	Stimulating myocardial PI3K-Akt pathway; inhibiting the activation of caspase 3 and Bax/Bcl 2 ratio	Chen et al. (2020b)
	GLXB	*Trichosanthes kirilowii. [Cucurbitaceae], Allium chinense [Amaryllidaceae]* and *Banxia Pinellia ternata [Araceae]*	Increasing mRNA and protein levels of PI3K, Akt and eNOS	Fu et al. (2020)
	GGD	*Neolitsea cassia. [Lauraceae]* and *Glycyrrhiza glabra L. [Fabaceae]*	Upregulating Bcl-2, PPAR-α, and PPARγ; downregulating Bax, caspase-3 and caspase-9	Gao et al. (2019)
	DGAD	*Aconitum lethale. [Ranunculaceae]* and *Zingiber officinale [Zingiberaceae]*	Activating the PI3K/Akt/GSK-3β signaling	Xie et al. (2021)
	QLQX	*Astragalus mongholicus [Fabaceae], Panax ginseng [Araliaceae], Salvia miltiorrhiza Bunge [Lamiaceae], Descurainia Sophia [Brassicaceae], Alisma plantago-aquatica subsp. orientale [Alismataceae], Polygonatum odoratum [Asparagaceae], Neolitsea cassia. [Lauraceae], Carthamus tinctorius L. [Asteraceae], Periploca sepium Bunge [Apocynaceae]* and *Citrus aurantium L. [Rutaceae]*	Elevating the Bcl-2 expression, the ratios of phospho-Akt/Akt and phospho-GSK3β/GSK3β; declining the expressions of Bax, Cyt-c, Apaf-1, cleaved caspase-9 and cleaved caspase-3	Zhao et al. (2019)
	QSG	*Angelica sinensis (Oliv.) Diels [Apiaceae], Lonicera japonica Thunb. [Caprifoliaceae], Scrophularia ningpoensis Hemsl. [Scrophulariaceae]* and *Glycyrrhiza uralensis Fisch. ex DC. [Fabaceae]*	Increasing Bcl-2 and caspase-3/12; reducing the expressions of Bax and cleaved caspase-3/12	Zhang et al. (2020a)
Injection	SLI	*Salvia miltiorrhiza Bunge [Lamiaceae]* and ligustrazine injection	Inhibiting the activation of caspase-3 and Bax/Bcl-2 ratio	Huang et al. (2016)
	DHI	*Salvia miltiorrhiza Bunge [Lamiaceae]* and *Carthamus tinctorius L. [Asteraceae]*	Preventing NF-κB nuclear translocation	Chen et al. (2019a)
	SMI	*Panax ginseng C.A.Mey. [Araliaceae]* and *Ophiopogon japonicus (Thunb.) Ker Gawl. [Asparagaceae]*	Increasing PI3K and p-Akt expression	Li et al. (2020e)
		*Panax ginseng C.A.Mey. [Araliaceae], Ophiopogon japonicus (Thunb.) Ker Gawl. [Asparagaceae]* and *Schisandra chinensis (Turcz.) Baill. [Schisandraceae]*	Inhibiting the cleaved caspase-3 protein and Bax/Bcl-2 ratio	Liu et al. (2018a)
	SXNI	*Ginkgo biloba L. [Ginkgoaceae]*	Inhibiting the cleaved caspase-3 protein and Bax/Bcl-2 ratio	Li et al. (2021)
	HJTI	*Cardiospermum halicacabum L. [Sapindaceae]*	Decreasing the levels of cleaved caspase-3; increasing the Bcl-2/Bax ratio	Zhao et al. (2021a)
Effective constituents	Tanshinone 2A	——	Increasing the expression of Bcl-2 and Bak; reducing the expression of caspase-3, cyt-c and Apaf-1	Tai et al. (2021), Fangm et al. (2021)
	Sal-A	——	Decreasing tunnel-positive cells and Bax	Zhou et al. (2020)
	Sal-B	——	Activating PI3K/Akt pathway; Inhibiting HMGB1 expression	Li et al. (2020a)
	G-Rg1	——	Improving energy metabolism to inhibiting myocardial apoptosis	Li et al. (2018a)
	G-Rb3	——	Protecting the integrity of mitochondrial membrane to prevent apoptosis	Sun et al. (2019)
	6-Gingerol	——	Inhibiting caspase-3 activation; upregulating the expression of PI3K, p-Akt and Akt	Lv et al. (2018)
	Salidroside	——	Downregulating the expression levels of TNF-α, Bax, cleaved caspase-12, caspase-3; upregulating the expression of Bcl-2, VEGF, Akt and eNOS	Chen et al. (2019a), Sun et al. (2018)
	Paeoniflorin	——	Increasing Bcl-2 protein level; decreasing caspase-3, Bax, p-ERK, p-JNK and p-p38	Wu et al. (2020)
	Total paeony glycoside	——	Upregulating the expression of pro-caspase-3 and Bcl-2; downregulating cleaved-caspase-3, Bcl-2-associated X protein, PI3K and Akt expression	Shen et al. (2018)
	Paeonol	——	Upregulating Bcl-2 protein expression; downregulating the cleaved caspase-8, caspase-9, caspase-3 and PARP protein expression	Tsai et al. (2021)
	Ginkgolide B	——	Repressing Bax/Bcl-2 and cleaved caspase-3	Ren et al. (2020)

### 2.3 Treatment of traditional Chinese medicine on cerebral ischemia

#### 2.3.1 Chinese herbal compound prescriptions

Coincidentally, magnanimous TCM prescriptions and their active components can also assuage ischemic brain injury by inhibiting apoptosis (Table 2). *Conioselinum anthriscoides* “*Chuanxiong*”’ *[Apiaceae]* and *Paeonia lactiflora Pall.* [*Paeoniaceae*] (CA-PL) drug pair could ameliorate cerebral ischemia by inhibiting the inflammatory reaction and apoptosis in MCAO rats on account of down-regulating the expression of caspase-3 and caspase-12 (Gu et al., 2018). In addition, Zhou et al. (2021b) found that CA-PL attenuates cerebral ischemic injury via inducing cell proliferation and differentiation, decreasing the expression of inflammatory factors and antagonizing neuronal apoptosis. It is worth mentioning that the combination use of CA and PL exerted more significant anti-inflammatory and anti-apoptotic effects on ischemic stroke than alone. The treatment of *Astragalus mongholicus Bunge* [*Fabaceae*] AM-CA protected rat brain microvascular endothelial cells from oxygen and glucose deprivation reperfusion (OGD/R)-induced apoptosis on account of prominently decreasing cellular apoptosis and promoted cell viability (Tang et al., 2021). In addition, a study showed the effect of co-administration of *Eleutherococcus senticosus* (*Rupr. & Maxim.*) *Maxim. [Araliaceae]* (ES) and *Gastrodia elata Blume [Orchidaceae]* (GE) could improve neuronal injury and prevent CIRI and neuronal apoptosis by attenuating oxidative stress and inflammation (Lin et al., 2021). *C. tinctorius L. [Asteraceae]* (CT) exhibited a protective effect on cerebral I/R by reducing cerebral ischemic injury and improving neurological function distinctly. In addition, CT can also reduce the degree of apoptosis in brain tissue and remarkably reduce the expression of matrix metalloproteinases, followed by the expression of tissue inhibitor of metalloproteinases one protein (Chang et al., 2020). Cheng et al. (2020) found that treatment of *Angelica sinensis* (*Oliv.*) *Diels [Apiaceae]* (AS) had neuroprotective effects on I/R injury by activating p38 MAPK. It has been found that a high dose of *Blumea balsamifera* (*L.*) *DC. [Asteraceae]* (BB) can notably reduce the expression levels of Apaf-1, Bad, and Caspase-3 and enhance the Bcl-2 expression level (Zhang et al., 2021a). The mechanism may be to improve the function of the neurovascular unit through anti-apoptosis and anti-inflammation and to maintain the stability of the blood-brain barrier and tight junctions (Dong et al., 2018). Dong et al. (2019) found that the aqueous extract of SM upregulated the expression of Bcl-2 and down-regulated the expression of Bax in the MCAO/R mouse model (Dong et al., 2018).

**TABLE 2:** The involved mechanisms of representative TCM prescriptions in the treatment of cerebral ischemia.

Categories	TCM	Composition	Mechanisms	References
Drug pairs	CA-PL	*Conioselinum anthriscoides [Apiaceae] and Paeonia lactiflora Pall. [Paeoniaceae]*	Downregulating the expression of caspase-3 and caspase-12; increasing the expression of Ras, ErbB and VEGF	Gu et al. (2018), Zhou et al. (2021)
Extract	CT	*Carthamus tinctorius L. [Asteraceae]*	Reducing the expression of MMP-9, Bax/Bcl-2 and caspase-3	Chang et al. (2020)
	AS	*Angelica sinensis (Oliv.) Diels [Apiaceae]*	Increasing p-p38 MAPK, Cyt-c, and cleaved caspase-3 expression	Cheng et al. (2020)
	BB	*Blumea balsamifera (L.) DC. [Asteraceae]*	Reducing the gene and protein levels of Apaf-1, Bad and caspase-3; increasing the expression of Bcl-2	Zhang et al. (2021a)
	Aqueous extract of SM	*Salvia miltiorrhiza Bunge [Lamiaceae]*	Increasing the Bcl-2/Bax ratio	Meng et al. (2018)
Formulas	BYHWD	*Astragalus mongholicus Bunge [Fabaceae], Angelica sinensis (Oliv.) Diels [Apiaceae], Paeonia lactiflora Pall. [Paeoniaceae], Conioselinum anthriscoides [Apiaceae], Carthamus tinctorius L. [Asteraceae], Prunus persica (L.) Batsch [Rosaceae]* and *Galanthus nivalis L. [Amaryllidaceae]*	Upregulating the expression of p-PI3K, p-Akt, and p-Bad and JAK2/STAT3/Cyclin D1 signaling cascades	Zhang et al. (2018), Shen et al. (2020a), Chen et al. (2020a)
	LTC	Draconis phenols extract	Inhibiting the cleavage of PARP, caspase-3 and caspase-9	Pan et al. (2021), Pan et al. (2018)
	TXL	*Panax ginseng C.A.Mey. [Araliaceae], Glycyrrhiza glabra L. [Fabaceae] and Tragia involucrata L. [Euphorbiaceae], Paeonia lactiflora Pall. [Paeoniaceae], Cicada Slough, Physalis cordata Houst. ex Mill. [Solanaceae], Centipeda minima (L.) A.Braun & Asch. [Asteraceae], Sandalwood Incense, Dalbergia odorifera T.C.Chen [Fabaceae], Boswellia sacra Flück. [Burseraceae], sour Jujube Kernel (fried) a*nd Cinnamomum camphora (L.) J.Presl [Lauraceae]	Decreasing Bax and cleaved caspase-3	Cheng et al. (2017)
	BNFY	Developed from Buyang Huanwu decoction	Downregulating TLR4, NF-κB, p-p38 MAPK expression; upregulating p-Akt expression	Gao and Gu (2020)
	NTF	*Astragalus mongholicus Bunge [Fabaceae]*,*Conioselinum anthriscoides [Apiaceae]* and *Pheretima, Bombyx Batryticatus*	Increasing the Bcl-2/Bax ratio	Yang et al. (2021b)
	GLGZD	*Trichosanthes kirilowii Maxim. [Cucurbitaceae], Neolitsea cassia (L.) Kosterm. [Lauraceae], Paeonia lactiflora Pall. [Paeoniaceae], Glycyrrhiza glabra L. [Fabaceae], Curcuma longa L. [Zingiberaceae]* and *Ziziphus jujuba Mill. [Rhamnaceae]*	Reducing expression of PAR; increasing expression of mitochondrial AIF and Endo G	Nan et al. (2020)
	QNDP	*Gardenia jasminoides J.Ellis [Rubiaceae], Panax notoginseng (Burkill) F.H.Chen [Araliaceae]* and *Cinnamomum camphora (L.) J.Presl [Lauraceae]*	Inhibiting NLRP3 inflammasome signaling pathway	Fu et al. (2020)
	AGNHW	*Cinnamomum camphora (L.) J.Presl [Lauraceae], Coptis chinensis Franch. [Ranunculaceae], Bombyx Batryticatus, Saposhnikovia divaricata (Turcz. ex Ledeb.) Schischk. [Apiaceae], Cinnamomum camphora (L.) J.Presl [Lauraceae], Bovis Calculus, Abelmoschus moschatus Medik. [Malvaceae], Tragia involucrata L. [Euphorbiaceae], Pinellia ternata (Thunb.) Makino [Araceae], Trichosanthes kirilowii Maxim. [Cucurbitaceae], Ribes uva-crispa subsp. uva-crispa [Grossulariaceae], Scutellaria baicalensis Georgi [Lamiaceae], Wurfbainia villosa (Lour.) Skornick. & A.D.Poulsen [Zingiberaceae]* and refined honey	Inhibiting of Bax/Bcl-2 ratio and caspase-3 level	Zhang et al. (2021b), Wang et al. (2014)
	DSS	*Angelica sinensis (Oliv.) Diels [Apiaceae], Paeonia lactiflora Pall. [Paeoniaceae], Poria cocos, Atractylodes macrocephala Koidz. [Asteraceae], Alisma plantago-aquatica subsp. orientale (Sam.) Sam. [Alismataceae]* and *Conioselinum anthriscoides [Apiaceae]*	Downregulating the expression of cleaved caspase-3 and Bax; up-regulating Bcl-2	Luo et al. (2021)
Injection	DSCXQ	*Salvia miltiorrhiza Bunge [Lamiaceae]* and *Conioselinum anthriscoides [Apiaceae]*	Inhibiting the expression of Sphk1, S1PR1, CD62P, Bax and cleaved caspase-3; increasing the level of Bcl-2	Zhou et al. (2021b)
	XNJ	*Abelmoschus moschatus Medik. [Malvaceae], Curcuma longa L. [Zingiberaceae], Cinnamomum camphora (L.) J.Presl [Lauraceae]* and *Gardenia jasminoides J.Ellis [Rubiaceae]*	Activating PI3K/Akt/eNOS signaling	Zhang et al. (2018b)
	SXNI	*Ginkgo biloba L. [Ginkgoaceae]. extract*	Inhibiting Bax/Bcl-2 and blocking caspase-3 activation	Li et al. (2021)
Effective constituents	AS-IV	——	Inhibiting cleaved caspase-3 and CaSR, Bax/Bcl-2 ratio and p62; increasing the expression of LC3II/LC3I	Du et al. (2021), Zhang et al. (2019a), Xu et al. (2020a)
	HSYA	——	Inhibiting NF-κB, caspase-3 and MAPK signal pathway	Yang et al. (2020)
	AME-PNS	——	Inhibiting JNK signal transduction, reducing cyt-c, caspase-9 and caspase-3	Huang et al. (2017), Xu et al. (2020b)
	AS-IV - HSYA	——	Reducing the expression of PHLPP-1; activating Akt signaling	Cao et al. (2020)
	HSYA- HSYB	——	Decreasing the expression of Bax/Bcl-2 ratio	Fangma et al. (2021)
	Salvianolate	——	Inhibiting the level of ROS and the caspase-3 signaling pathway	Luan et al. (2020)
	Baicalein	——	Decreasing the expression of caspase-3 and Bax/Bcl-2 ratio	Yang et al. (2019)
	Baicalin	——	Activating AMPK pathway	Li et al. (2018a)
	Baicalin/Geniposide	——	Decreasing TNF-α, IL-1β, NF-κB and pNF-κB	Li et al. (2021)
	Glucosides of paeony	——	Reducing cleaved caspase-3 and Bax/Bcl-2 ratio	Li et al. (2020b)
	Paeoniflorin	——	Inhibiting Bax, Bad, caspase-3 and caspase-9 expression; increasing Bcl-2 and Bcl-xL expression	Zhang et al. (2016), Chen et al. (2017)
	Salidroside	——	Activating PI3K/Akt pathway and complement system	Zhang et al. (2018c), Wang et al. (2020a)
	Asiaticoside	——	Inhibiting NOD2/MAPK/NF-κB signaling pathway	Zhang et al. (2020b)
	Harpagide and SN	——	Decreasing GRP78, caspase-12 and CHOP expression	Wang et al. (2020b), Chen et al. (2020c)
	Ginsenosides	——	Inhibiting NF-κB transcriptional activity and the expression of IL-1β, TNF-α and IL-6	Cheng et al. (2019a)
	Ginkgolide K	——	Decreasing the protein expression levels of p-p38, p-JNK, p-p53, p-c-Jun and the expression levels of Bcl-2, Bax, cleaved caspase-9 and caspase-3	Tao et al. (2017), Liu et al. (2018a)
	Ginkgetin	——	Downregulating the levels of cleaved caspase-3 and Bax; upregulating the level of Bcl-2	Tian et al. (2019)

Buyang Huanwu decoction (BYHWD) is famous for treating ischemic stroke and the resulting behavioral symptoms of ischemia (Zhang et al., 2018a), including neurological impairment and spatial learning and memory function. Evidence indicated that it could remarkably increase microvessel density and cerebral blood flow in the ischemic penumbra (Shen et al., 2020). In addition, it can activate PI3K/Akt/Bad and Jak2/Stat3/Cyclin D1 signaling pathways to exert a neuroprotective role (Chen et al., 2020b). For the Longxue Tongluo capsule (LTC), Pan et al. observed that it effectively inhibited the apoptosis of PC12 cells induced by OGD/R to suppress the cleavage of PARP, caspase-3, and caspase-9 (Pan et al., 2021). Further studies have shown that LTC can protect human umbilical vein endothelial cells from OGD/R damage by inhibiting the activation of the mitochondrial-related apoptosis pathway (Pan et al., 2018). It was reported that Tongxinluo (TXL) also has anti-apoptotic cerebral ischemia protection by means of the Cx43/Calpain II/Bax/caspase-3 pathway, contributing to the prevention and therapy of I/R injury (Cheng et al., 2017). Additionally, Naotaifang (NTF) was detected to increase the expression level of Bcl-2 and decrease the expression level of Bax (Yang et al., 2021a). To further study the effect of Gualou Guizhi decoction (GLGZD) on MCAO I/R injury, the involved mechanism was mainly relevant to regulating PARP-1/AIF signal pathway (Nan et al., 2020). Fu et al. illustrated that Qingnao-dropping pills (QNDP) could notably ameliorate cerebral ischemic injury and neurological function by astricting apoptosis in rats with MCAO injury (Fu et al., 2020). As a prestigious formula in treating cerebral diseases, An Gong Niu Huang Wan (AGNHW) could stimulate the amount of Nissl, NeuN, and DCX positive cells by lowering the number of Tunel positive cells, the Bax/Bcl-2 ratio, and caspase-3 level (Wang et al., 2014; Zhang et al., 2021b). A recent study has shown that the ethanol extract of Danggui Shaoyao San (DSS) has a neuroprotective effect on ischemic brain injury by down-regulating the expression level of cleaved-caspase-3 and Bax, while up-regulating the expression level of Bcl-2 (Luo et al., 2021). In addition to the above TCM formula, some injections also have neuroprotective effects on the cerebral ischemic disease by regulating apoptosis. For example, Danshen Chuanxiongqin injection (DSCXQ) remarkably functioned in the prevention of neuronal apoptosis via inhibiting the expression of CD62P, Sphk1, S1PR1, Bax/Bcl-2 and cleaved caspase-3 (Zhou et al., 2021c). Both *in vivo* and *in vitro* evidence suggested that the antineuronal apoptotic effect of Xingnaojing injection (XNJ) contributed to the improvement of ischemic brain injury. The mechanism may be related to the activation of the PI3K/Akt/eNOS signal pathway (Zhang et al., 2018b). Besides, Shuxuening injection (SXNI) inhibited hippocampal neuronal apoptosis by regulating Bax/Bcl-2 and inhibiting caspase-3 activation (Li et al., 2021).

### 2.4 Active ingredients of traditional Chinese medicine

Astragaloside IV (AS-IV) is an active ingredient from Chinese herbal medicine. Many studies have shown that AS-IV alleviated neurological functional score and cerebral infarction volume via inhibiting CIRI-induced expression of cleaved caspase-3 and Ca^2+^-sensing receptor (CaSR) (Xu et al., 2020a; Hou et al., 2020; Du et al., 2021). In addition, AS-IV promoted the viability of HT22 cells subjected to OGD/R *via* decreasing the expression of p62, while enhancing the ratios of Bcl-2/Bax and LC3II/LC3I (Zhang et al., 2019a), suggesting its potential regulation of apoptosis and autophagy. Yang et al. (2020) found that blocking glycogen synthase kinase-3β can partially reverse the protective effect of HSYA yellow A (HSYA) on I/R by regulating NF-κB and caspase-3. Combining two compounds has also been widely used in cerebral ischemic diseases. The protective effect of *A. mongholicus Bunge [Fabaceae]* extract (AME) combined with *Panax notoginseng* saponins (PNS) on the cerebral ischemic injury was enhanced. Its mechanism may be related to inhibiting the JNK signal transduction-related mitochondrial apoptosis pathway (Xu et al., 2020b; Hou et al., 2020). Huang et al. further argued that the PNS and AME-PNS could significantly reduce apoptosis by down-regulating the level of phosphorylated C-6-n-terminal kinase 1/2 (p-JNK1/2), Cyt-c, caspase-9, and caspase-3 (Huang et al., 2017). Cao et al. found that AS-IV combined with HSYA reduced the cell loss caused by OGD through inhibiting apoptosis (Cao et al., 2020). *In vivo* experiments exhibited that the amelioration of infarct volume and neurological function by HSYA and HSYB was caused by apoptosis inhibition (Fangma et al., 2021). Salvianolic acid A subdued neuronal apoptosis in rats MCAO/R by repressing the caspase-3 signal pathway (Luan et al., 2020). As flavonoids isolated from *Scutellaria baicalensis Georgi [Lamiaceae]*, the therapeutic effect of baicalein and baicalin on subacute brain injury induced by MCAO in rats was related to increasing the Bcl-2/Bax rate and decreasing the level of caspase-3 by activating AMPK signal (Yang et al., 2019). Miraculously, the combination of baicalin and geniposide reduced the apoptosis *via* the activation of microglial cells induced by OGD/R in a dose-dependent manner (Li et al., 2021). Paeoniflorin is one of the important active ingredients from *P. lactiflora Pall. [Paeoniaceae]* presents a neuroprotective effect. The results showed that paeoniflorin significantly decreased infarct volume by confronting Ca^2+^ overload-induced neuronal apoptosis (Wu et al., 2020). According to the report, salidroside can be used as an effective anti-apoptotic neuroprotective agent protecting ischemic stroke by regulating the PI3K/Akt mediated apoptotic pathway (Zhang et al., 2018c) and the complement system (Wang et al., 2020c). Zhang et al. found that asiaticoside can reverse the apoptotic process through blocking the NOD2/MAPK/NF-κB signal pathway, which could reduce nerve damage, brain edema, infarct size, inflammation, and oxidative stress in the CIRI model (Zhang et al., 2020b). Some studies announced that the active components of *Scrophularia ningpoensis Hemsl. [Scrophulariaceae]* (SN) have a protective effect of anti-apoptosis on ischemic brain injury. It has been proved that harpagide and iridoid glycosides of SN had a neuroprotective effect by decreasing GRP78, caspase-12, and CHOP gene and protein expression (Wang et al., 2020d). Iridoid glycosides possessed a protective effect on CIRI in brain tissue by inhibiting ERS-triggered apoptosis (Chen et al., 2020c). Cheng et al. proved that ginsenosides could reduce apoptosis by increasing mitochondrial membrane potential and inhibiting the ROS production in the cobalt chloride-simulated PC12 cells hypoxia injury (Cheng et al., 2019a). Some researchers proved that bilobalide, an active component of *Ginkgo biloba L. [Ginkgoaceae]*, could significantly reduce apoptosis and autophagy to promote angiogenesis after CIRI (Zheng et al., 2018). In addition, Ginkgolide K could protect the nervous system from stimulating cerebral ischemia *in vitro*, which was achieved by inhibiting p38 and the JNK-activated mitochondrial apoptosis pathway (Tao et al., 2017; Liu et al., 2018b). Besides, Ginkgetin could significantly reduce the amounts of apoptotic cells via inhibiting apoptosis through activating the PI3K/Akt/mTOR signal pathway in a dose-dependent manner (Tian et al., 2019).

## 3 Discussion

According to TCM theory, different diseases could be treated with the same method or herbs since the key point of therapy was neither the cause nor the disease symptom but the identification of the same pathogenesis for different diseases, which reflects the holistic view of TCM in the treatment of diseases (Zhai et al., 2020). There are great similarities in the pathogenesis and processes of cardiovascular and cerebrovascular disease in TCM, which provides a favorable pathological basis for TCM to treat these two different diseases.

Studies mentioned above illustrate that apoptosis plays an important role in cerebral ischemia, myocardial ischemia, and reperfusion injury. The inhibition of apoptosis by TCM is multi-channel and multi-target, which involves many apoptosis pathways, including the mitochondria apoptosis, death receptor apoptosis, and endoplasmic reticulum apoptosis pathway. It has been reported that apoptosis regulated by ER stress can inhibit MIRI by activating the PI3K/Akt signal pathway (Li et al., 2018a). A previous study has shown that Tong mai Yang Xin pill (TMYX) inhibited myocardial no-reflow after I/R injury via activating the PI3K/Akt/eNOS pathway, modulating apoptosis, up-regulating NO activity, and relaxing coronary microvessels (Chen et al., 2020a). Another research indicated that AS-IV could improve cardio-protection and angiogenesis after myocardial infarction by activating PTEN/PI3K/Akt signaling pathway (Cheng et al., 2019b). Moreover, Buyang Huanwu decoction, XNJ injection, Tao Hong Si Wu decoction, TXL and tanshinol were found to take neuroprotective effect by activating the pi3K-Akt pathway. (Li et al., 2015; Yu et al., 2016; Zhang et al., 2018a; Zhang et al., 2019b; Shen et al., 2020). Similarly, current reports suggest that targeting the p53 pathway is a potential neuroprotective strategy against the ischemic injury (Nijboer et al., 2011). The therapeutic effect of the Huoxin pill, Xiao-Xu-Ming decoction and XNJ injection protect ischemic tissue through stimulating p53 transcriptional activity (Lan et al., 2014; Wei et al., 2015; Shen et al., 2020). These studies show that the apoptosis pathway and molecular mechanism involved in cerebral and myocardial ischemia have common characteristics, which provide a molecular mechanism for TCM to treat ischemic diseases.

This review showed the drug pairs of salvian-safflower injection, salvian-Chuanxiong injection, and Danhong injection and their active components salvianolic acid A, salvianolic acid B, and tanshinol were the main TCM used to treat myocardial ischemia by inhibiting apoptosis. Furthermore, drugs such as *P. ginseng C.A.Mey. [Araliaceae], A. mongholicus Bunge [Fabaceae], C. tinctorius L. [Asteraceae]*, and *C. anthriscoides* “*Chuanxiong*” *[Apiaceae]* also show great therapeutic potential for myocardial ischemia. *A. mongholicus Bunge [Fabaceae]-Foeniculum vulgare Mill. [Apiaceae], F. vulgare Mill. [Apiaceae]* extract and its injection showed superior efficacy in cerebral ischemia treatment. *C. tinctorius L. [Asteraceae]* extract and its active components, hydroxyl safflower A and hydroxyl safflower B, played a therapeutic role in cerebral ischemia by inhibiting apoptosis. The herbs, including SM, *A. mongholicus Bunge [Fabaceae], C. anthriscoides* “*Chuanxiong*” *[Apiaceae], A. sinensis (Oliv.) Diels [Apiaceae], C. tinctorius L. [Asteraceae],* and *P. ginseng C.A.Mey. [Araliaceae],* and their active ingredients, have potential therapeutic effects on both myocardial and cerebral ischemia by inhibiting apoptosis. In addition, *G. biloba lactone, salidroside*, and *P. notoginseng saponins* have therapeutic effects on myocardial ischemia and cerebral ischemia. The main components are saponins, phenolic acids, esters, and flavonoids. These results showed that certain botanical drugs used in TCM show effects relevant for treating myocardial ischemia and cerebral ischemia. In recent years, TCM has made considerable progress in preventing and treating cardiovascular and cerebrovascular diseases. Herein, we analyzed the role of apoptosis in ischemic cardiovascular and cerebrovascular diseases. The result indicated that these two diseases’ mechanisms and targets might be closely related to apoptosis. TCM has been considered effective in treating ischemic cardio-cerebrovascular diseases by inhibiting apoptosis. Notably, drugs for treating these two diseases have common characteristics, mainly to tonifying qi and promoting blood circulation. The active components mainly include saponins, phenolic acids, esters, and flavonoids. But regretfully, we mainly concentrated on the therapeutical effect of TCM in treating ischemic diseases from the prospective of anti-apoptosis in this review. But as a matter of fact, the protection effect of TCM on the ischemia disease is also involved in many other mechanisms, such as inhibiting oxidative stress, pyroptosis and autophagy (Wang et al., 2020e; Zhang et al., 2021c; Dambrova et al., 2021). Thus, more clinical investigations are urgently needed to illustrate the pharmacodynamic substance basis of TCM and their potential therapeutic targets in ischemic cardiovascular and cerebrovascular disease treatment.
